# Low Immunity to Measles and Rubella among Female Guest Workers, Northern Mariana Islands

**DOI:** 10.3201/eid1511.081267

**Published:** 2009-11

**Authors:** Vicki Stambos, Jean-Paul Chaine, Heath Kelly, Mariana Sablan, Michaela Riddell

**Affiliations:** Victorian Infectious Diseases Reference Laboratory, Melbourne, Victoria, Australia (V. Stambos, H. Kelly, M. Riddell); Department of Public Health, Saipan, Commonwealth of the Northern Mariana Islands (J.-P. Chaine, M. Sablan)

**Keywords:** Vaccines, immigration, measles, rubella, immunization, Northern Mariana Islands, guest workers, viruses, letter

**To the Editor:** The Commonwealth of the Northern Mariana Islands (CNMI), a group of northern Pacific islands in political union with the United States, was exempt from US labor laws until 2007. This exemption attracted business opportunities, which led to a high demand for guest workers. The Centers for Disease Control and Prevention advises the US Citizenship and Immigration Services of vaccination requirements for those applying for immigration and work visas before the applications are approved (*1*). Since 1996, all applicants born after 1957 and >12 months of age have been required to provide evidence of completed vaccination against, or of immunity to, measles, mumps, and rubella viruses. Those unable to provide such evidence must receive at least 1 dose of the vaccines recommended by the US Advisory Committee on Immunization Practices before visa approval. The Committee also advises applicants to receive additional doses of the required vaccines after arrival in the Mariana Islands. We aimed to determine the proportion of CNMI guest workers who were immune to measles and rubella by testing a convenience sample of serum collected during September and October 2006. However, procedures for validating the vaccination status for our sample population are unknown. Given our results, it appears that validation procedures of immunity status in guest workers or immigrants to the United States were suboptimal at the time of this study.

Serum samples from 210 female workers from 17 through 51 years of age were collected opportunistically when, as a requirement for annual contract renewal, the workers came to the Department of Public Health, Saipan, CNMI. Approximately 70% of these guest workers were from the People’s Republic of China and the Philippines and were employed in the garment and hospitality industries (*2*). We estimated that a minimum sample size of 196 would provide a precision estimate of 5% based on an anticipated proportion immune of 85% (actual study size was 210 samples). Informed consent was obtained from all participants. Serum samples, with identifying information removed, were shipped to the Victorian Infectious Diseases Reference Laboratory, Melbourne, Australia.

Immunoglobulin G against measles and rubella was detected in serum by using Enzygnost ELISAs (Dade Behring, Deerfield, IL, USA) according to manufacturer’s instructions. For measles and rubella, samples with optical density (OD) values >0.2 (equivalent to 330 mIU/mL) indicated protective immunity and samples with OD values <0.1 were suggestive of no protection (*3*). Samples with OD values in the equivocal range (0.1–0.2) were retested, and the repeat result was recorded. Repeat equivocal results were classified as not protected. Data were analyzed by using STATA version 8.2 (Stata Corp., College Station, TX, USA). Exact binomial 95% confidence intervals were calculated. Proportions of guest workers immune to measles and rubella, by age group and country of origin, were assessed by Fisher exact test and χ^2^ statistics.

The proportion of Chinese guest workers immune to measles (115/154, 74.7%) and rubella (131/154, 85.1%) was lower than the proportion immune of all other workers combined (56/56, 100% and 50/56, 89.3%, respectively), but the difference was only significant for measles (Table). When compared with Chinese workers of all other ages, Chinese workers 20–34 years of age were significantly less likely to be protected against measles (69.3% vs. 89.7%; p = 0.01). No significant differences were found in the proportion of guest workers immune to rubella by age group (p = 0.70) or country of origin (p = 0.43).

**Table:** Proportion of guest workers immune to measles and rubella by ethnicity and age group, Northern Mariana Islands, September–October 2006*

Age group, y	Proportion immune						
	Guest workers from China				Guest workers from other countries†		
	Total no. tested	No. (%) measles, 95% CI	No. (%) rubella, 95% CI		Total no. tested	No. (%) measles, 95% CI	No. (%) rubella, 95% CI
15–19	18	17 (94.4), 72.7–99.9	14 (77.8), 52.4–93.6		0	–	–
20–24	56	41 (73.2),59.7–84.2‡	49 (87.5), 75.9–94.8		1	1 (100), 2.5–100§	1 (100), 2.5–100§
25–29	40	25 (62.5), 45.8–77.3‡	36 (90.0), 76.3–97.2		10	10 (100), 69.2–100§	10 (100), 69.2–100§
30–34	18	13 (72.2), 46.5–90.3‡	15 (83.3), 58.6–96.4		9	9 (100), 66.4–100§	8 (88.9), 51.8–99.7
35–39	17	14 (82.4), 56.6–96.2	13 (76.5), 50.1–93.2		16	16 (100), 79.4–100§	15 (93.8), 69.8–99.8
40–44	2	2 (100), 15.8–100§	2 (100), 15.8–100§		14	14 (100), 76.8–100§	11 (78.6), 49.2–95.3
>45	2	2 (100), 15.8–100§	1 (50.0), 1.3– 98.7		6	6 (100), 54.1–100§	5 (83.3), 35.9–99.6
Total	153¶	114 (74.5), 66.8–81.2#	130 (85.1), 78.3–90.2		56	56 (100), 93.6–100§	50 (89.3), 78.1–96.0

*CI, confidence interval. †Philippines (n = 48), Japan (n = 6), South Korea (n = 1), Thailand (n = 1). ‡Significant difference in measles immunity of Chinese workers 20–34 years of age compared with that of Chinese workers of other age groups; p = 0.01. §1-sided confidence interval. ¶The age of 1 guest worker was not provided by the referring laboratory. #Significant difference in measles immunity between workers from China and other countries; p<0.001.

A limitation of our work is that the sample may not be representative of the CNMI guest worker population overall. Only 27% of guest workers in the CNMI are from China, and 43% are from the Philippines (J.-P. Chaine, pers. comm., March 2008), whereas in our study 73% of guest workers were from China, and 23% were from the Philippines. Also, no men were recruited for the study yet men represent 19% of guest workers from China (J.-P. Chaine, pers. comm., March 2008), so our findings should not be extrapolated to this group.

China and the Philippines report 94% and 92% childhood immunization coverage with 1 measles vaccine, respectively (*4**,**5*). Similar to other reports of low measles immunity in young adult populations (*6*), this study identified young adult female workers from China as a group particularly susceptible to measles infection with >25% (39/154) unprotected. Neither country implemented rubella vaccination before 2006, and the immune profile for rubella reflects age-specific seroprevalence for endemic disease; >10% of these women remain susceptible to rubella during their potential childbearing years (*7*).

More than 8,000 female workers from China were in the CNMI during the period of this survey, and as many as 2,000 may have been susceptible to measles, which would have facilitated sustained transmission if the virus had been introduced. Several studies have shown that unvaccinated persons are clustered geographically or socially and may be at increased risk for measles or rubella outbreaks (*8**,**9*). These reports underscore the possible risk of virus spread in populations with low immunity in Saipan.
